# Mutations that prevent methylation of cohesin render sensitivity to DNA damage in *S. pombe*

**DOI:** 10.1242/jcs.214924

**Published:** 2018-07-06

**Authors:** Swastika Sanyal, Lucia Molnarova, Judita Richterova, Barbora Huraiova, Zsigmond Benko, Silvia Polakova, Ingrid Cipakova, Andrea Sevcovicova, Katarina Gaplovska-Kysela, Karl Mechtler, Lubos Cipak, Juraj Gregan

**Affiliations:** 1 Department of Chromosome Biology, MFPL, University of Vienna, Dr. Bohr-Gasse 9, 1030 Vienna, Austria; 2 Department of Genetics, Faculty of Natural Sciences, Comenius University in Bratislava, Ilkovicova 6, 842 15 Bratislava, Slovakia; 3 Department of Membrane Biochemistry, Institute of Animal Biochemistry and Genetics, Centre of Biosciences, Slovak Academy of Sciences, 84505 Bratislava, Slovakia; 4 Department of Genetics, Cancer Research Institute, Biomedical Research Center, Slovak Academy of Sciences, Dubravska cesta 9, 84505 Bratislava, Slovakia; 5 Research Institute of Molecular Pathology, Campus-Vienna-Biocenter 1, 1030 Vienna, Austria; 6 Advanced Microscopy Facility, Vienna Biocenter Core Facilities, Dr. Bohr-Gasse 3, 1030 Vienna, Austria

**Keywords:** Cohesin, DNA repair, Mitosis, Meiosis, *Schizosaccharomyces pombe*

## Abstract

The canonical role of cohesin is to mediate sister chromatid cohesion. In addition, cohesin plays important roles in processes such as DNA repair and regulation of gene expression. Mounting evidence suggests that various post-translational modifications, including phosphorylation, acetylation and sumoylation regulate cohesin functions. Our mass spectrometry analysis of cohesin purified from *Schizosaccharomyces pombe* cells revealed that the cohesin subunit Psm1 is methylated on two evolutionarily conserved lysine residues, K536 and K1200. We found that mutations that prevent methylation of Psm1 K536 and K1200 render sensitivity to DNA-damaging agents and show positive genetic interactions with mutations in genes encoding the Mus81–Eme1 endonuclease. Yeast two-hybrid and co-immunoprecipitation assays showed that there were interactions between subunits of the cohesin and Mus81–Eme1 complexes. We conclude that cohesin is methylated and that mutations that prevent methylation of Psm1 K536 and K1200 show synthetic phenotypes with mutants defective in the homologous recombination DNA repair pathway.

## Abstract (precis)

**Summary:** Cohesin methylation is a novel post-translational modification implicated in DNA repair in the fission yeast *S. pombe*.

## INTRODUCTION

The cohesin complex is a tripartite ring in which the Smc1 and Smc3 proteins (called Psm1 and Psm3, respectively, in the fission yeast *Schizosaccharomyces pombe*) are connected by their hinge domains on one side, and the kleisin subunit Rad21 (also called Scc1 or Mcd1) closes the ring by connecting the Smc1 and Smc3 head domains on the other side. Rad21 interacts with the fourth cohesin subunit, Scc3 (called Psc3 in fission yeast or SA/STAG in higher eukaryotes). In meiosis, the Rad21 and Psc3 subunits are replaced to a large extent by the meiosis-specific subunits Rec8 and Rec11, respectively.

The canonical role of cohesin is to mediate sister chromatid cohesion. Cohesion between sister chromatids is established during DNA replication and this process coincides with the acetylation of two lysine residues on Smc3 by the acetyltransferase Eco1/Ctf7 (Feytout et al., 2011; Rolef Ben-Shahar et al., 2008; Uhlmann and Nasmyth, 1998; Unal et al., 2008). To allow the segregation of sister chromatids, cohesion must be removed at the onset of anaphase. This removal involves phosphorylation of cohesin and cleavage of the kleisin subunit of the cohesin complex by separase (Alexandru et al., 2001; Uhlmann et al., 1999). Cohesin is also released from DNA in a separase-independent manner, which depends on phosphorylation of cohesin and involves the cohesin-associated protein Wapl (Waizenegger et al., 2000).

In addition to its role in mediating sister chromatid cohesion, cohesin is important for other processes, including DNA repair (Schar et al., 2004; Sjogren and Nasmyth, 2001; Strom et al., 2004; Unal et al., 2004). Cohesin accumulates at sites of double-strand DNA breaks (DSBs) where it presumably reinforces sister chromatid cohesion to facilitate repair by homologous recombination (Strom et al., 2004; Unal et al., 2004).

Homologous recombination is an important pathway for DSB repair and uses homologous DNA sequences as a template to facilitate DNA repair. DSB formation is followed by end-processing reactions in which DNA ends that flank DSBs are resected to expose 3′ single-stranded DNA (ssDNA) ends that are quickly bound by ssDNA-binding proteins. Mediator proteins such as Rad52, Rad55, Rad57 and Sfr1 then stimulate the replacement of ssDNA-binding proteins with Rad51 recombinases and stabilize Rad51–ssDNA filaments. These nucleoprotein filaments then catalyze the invasion of a homologous DNA template to form joint molecule intermediates that can be resolved by various enzymes, such as the Mus81–Eme1 endonuclease (Ranjha et al., 2018).

During S-phase, replication forks may stall when they encounter DNA lesions or natural replication barriers. Several proteins involved in homologous recombination, including the structure-specific endonuclease Mus81–Eme1 and the mediator protein Rad52, play an important role in the repair or rescue of blocked replication forks during S-phase (Doe et al., 2002; Lambert and Carr, 2013; Lorenz et al., 2009; Nguyen et al., 2015). However, the mechanisms by which homologous recombination proteins contribute to DNA replication remain poorly understood. Although it is well established that cohesin is important for the maintenance of genome integrity during S phase, we are only beginning to understand the molecular function of cohesin in the repair/rescue of blocked replication forks (Gelot et al., 2016; Tittel-Elmer et al., 2012).

Mounting evidence suggests that various post-translational modifications including phosphorylation, acetylation and sumoylation regulate the function of cohesin in DNA repair. Cohesin is sumoylated during the DNA damage response, and this modification is required for sister chromatid homologous recombination (Almedawar et al., 2012; McAleenan et al., 2012; Wu et al., 2012). The phosphorylation and acetylation of cohesin are required for DNA double-strand break-induced cohesion, and cohesin phosphorylation is also required for the intra-S checkpoint (Heidinger-Pauli et al., 2008, 2009; Kim et al., 2002; Watrin and Peters, 2009; Yazdi et al., 2002).

In recent years, protein methylation has been established as a major intracellular post-translational modification, but little is known about methylation of cohesin (Carlson and Gozani, 2016; Jung et al., 2008; Larsen et al., 2016; Olsen et al., 2016). In our current work, we show that, in the fission yeast *S. pombe*, the cohesin subunit Psm1 is post-translationally modified by methylation on two evolutionarily conserved lysine residues (K536 and K1200), and that Psm1 residues K536 and K1200, and possibly their methylation, are required for the proper functioning of the Mus81–Eme1-dependent DNA repair pathway.

## RESULTS AND DISCUSSION

### Psm1 is methylated on K536 and K1200

**Fig. 1.**
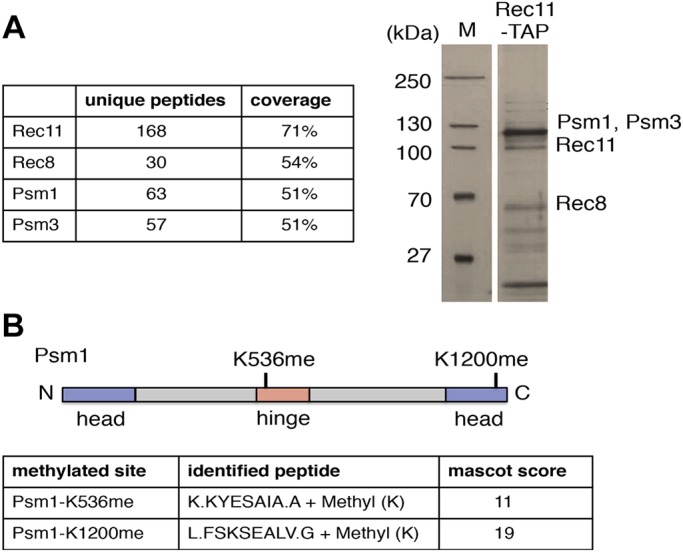
**Mass-spectrometry analysis reveals that Psm1 is mono-methylated on K536 and K1200.** (A) Cohesin subunits associated with Rec11–TAP were isolated by tandem affinity purification from meiotically induced *pat1-114/pat1-114 mat-Pc* cells harvested 2.5–3.5 h after meiosis induction. Purified proteins were separated on an SDS-PAGE gel and visualized by silver staining. Molecular mass markers (M) are shown on the left. Positions of Psm1, Psm3, Rec11 and Rec8 are indicated according to their predicted molecular mass. In parallel, samples were subjected to analysis by mass spectrometry. (B) Methylation sites identified on Psm1 and corresponding peptides are indicated.

To identify novel post-translational modifications on cohesin, we constructed functional tandem affinity purification (TAP)-tagged Rec11 (Rec11–TAP) according to our protocol described at http://mendel.imp.ac.at/Pombe_tagging (Cipak et al., 2009; Phadnis et al., 2015). We used a TAP protocol to purify Rec11–TAP together with other cohesin subunits (Psm1, Psm3 and Rec8) from meiotically induced *S. pombe* cells harvested around prophase I and analyzed the post-translational modifications by mass spectrometry (Fig. 1A; Fig. S1). We found that Psm1 was mono-methylated on two lysine residues (K536 and K1200) (Fig. 1B).

**Fig. 2.**
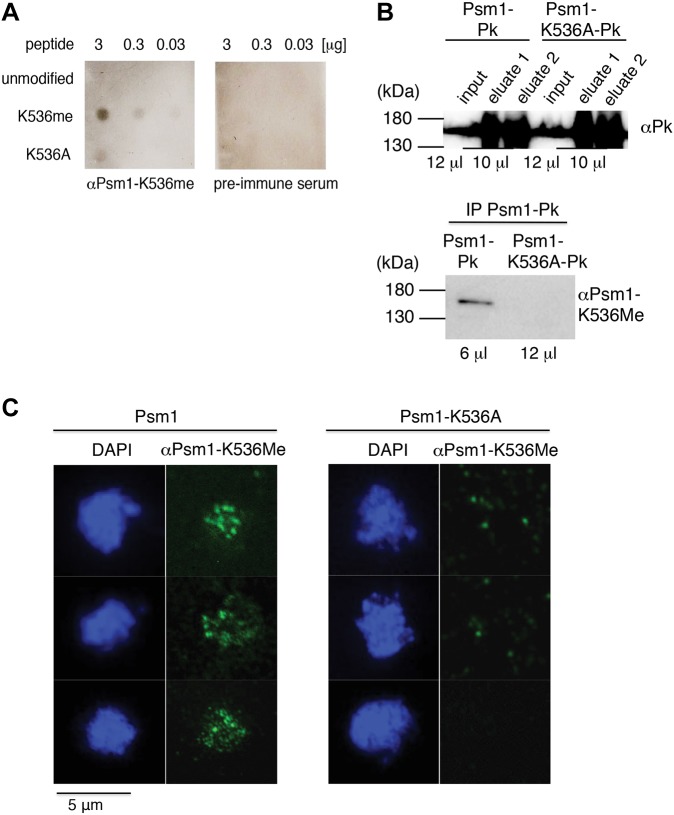
**Verification of Psm1-K536 methylation by anti-Psm1-K536me antibody.** (A) Specificity of antibody generated against Psm1-K536me. The indicated amounts of Psm1 peptide CTPTQKK(me)YESAIA carrying methylation on K536 (K536me), an unmodfied peptide CTPTQKKYESAIA (unmodified) or modified peptide CTPTQKAYESAIA (K536A) were spotted on PVDF membrane and probed with anti-Psm1-K536me antibody or pre-immune serum. (B) Psm1–Pk protein was immunoprecipitated (IP) from cycling wild-type cells (Psm1–Pk) or mutant cells carrying the *psm1^K536A^* mutation (Psm1-K536A–Pk). Samples were eluted in two steps and both were analyzed using anti-Pk antibody. Eluate 1 from each sample was analyzed with anti-Psm1-K536me antibody. The amount of loaded samples is indicated. The same protein extracts were analyzed on two different gels. (C) Nuclear spreads were prepared from cycling wild-type cells (Psm1) or mutant cells carrying the *psm1^K536A^* mutation (Psm1-K536A). Shown are selected nuclei after chromosome spreading, staining with DAPI and after immunolabeling with anti-Psm1-K536me antibody.

To exclude the possibility that this Psm1 methylation was an artifact introduced during the TAP purification or mass spectrometry analysis, we generated a polyclonal anti-Psm1-K536me antibody using methylated peptide [CTPTQKK(me)YESAIA] spanning the Psm1 methylation site K536me (Fig. 2A). We were not able to detect Psm1-K536me in total protein extracts (data not shown). However, upon immunoprecipitation of functional Pk-tagged Psm1 (Psm1–Pk) (Fig. S2B), our anti-Psm1-K536me antibody recognized a band of expected size in vegetative wild-type cells but not in mutant cells carrying a *psm1^K536A^* mutation that results in a replacement of the methylated lysine residue with an alanine residue, which can no longer be methylated (Psm1-K536A–Pk) (Fig. 2B). Although we cannot exclude the possibility that our anti-Psm1-K536me antibody binds to other epitopes and detects Psm1 regardless of its methylation status, given the specificity of this antibody for the modified peptide (Fig. 2A), it is likely that this band represents Psm1 methylated on K536.

Cohesin performs most of its functions on chromatin but there is also a soluble pool of cohesin present in the cell. To separate these two pools of cohesin, we prepared chromatin spreads from mitotically growing cells. Anti-Psm1-K536me antibody detected foci on spread chromatin from wild-type cells (Fig. 2C). These foci were much less prominent in spreads from mutant cells carrying the *psm1^K536A^* mutation, raising the possibility that chromatin-bound Psm1 is methylated on K536.

We conclude that Psm1 is methylated on lysine residues K536 and K1200. Our identification of methylation on Psm1 that co-purified with Rec11–TAP shows that methylated Psm1 is part of the cohesin complex. The fact that we were able to detect a specific signal using the anti-Psm1-K536me antibody on protein extracts and chromatin spreads from vegetative cells suggests that Psm1 is methylated on K536 not only in meiotic cells but also in mitotic cells, and that this methylation is likely present on chromatin-bound Psm1.

### A non-methylatable *psm1^K536A K1200A^* mutant is sensitive to DNA-damaging agents

**Fig. 3.**
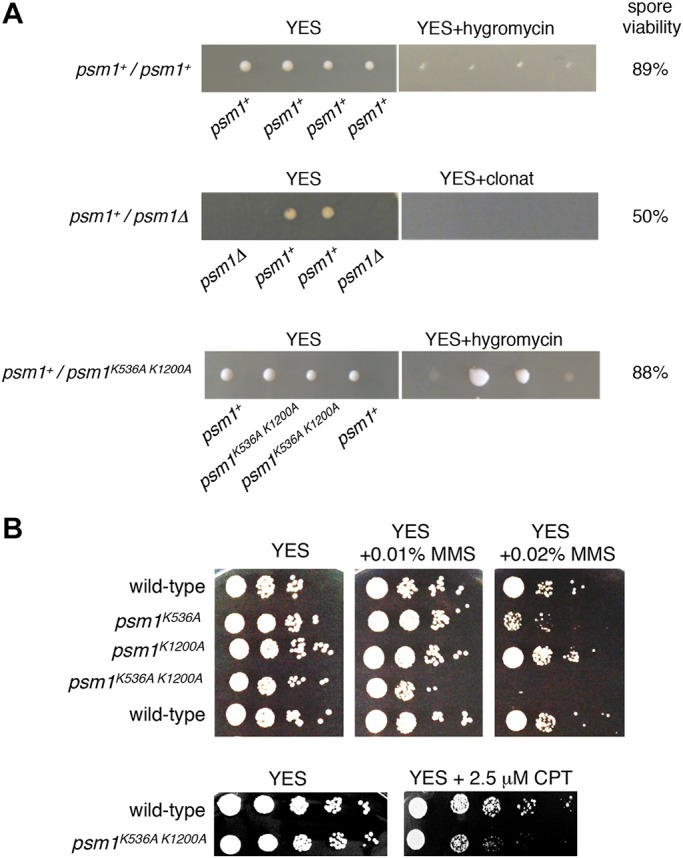
**The *psm1^K536A K1200A^* mutant is viable but sensitive to DNA-damaging agents.** (A) Tetrads were dissected from the indicated diploid strains on YES plates and after four days at 32°C, spore viability was determined. Colonies were replica-plated onto selective YES plates containing hygromycin B (200 mg/l) or clonat (100 mg/l). (B) Cells were grown on YES medium for 1 day, diluted in 5-fold steps, spotted onto YES plates containing the indicated amounts of MMS or CPT and incubated for 3 days at 30°C.

To test the potential functional significance of Psm1 methylation, we mutated the K536 and K1200 to alanine (*psm1^K536A^*, *psm1^K1200A^* and *psm1^K536A K1200A^*). Although replacing the lysine residue with alanine residues is a relatively large change, owing to different properties of these residues, alanine residue can no longer be methylated. After tetrad dissection of heterozygous diploid strains, we found that haploid *psm1^K536A K1200A^* mutant cells were viable and showed no apparent growth defect on YES plates (Fig. 3A,B).

To test whether Psm1 residues K536 and K1200 are important for repair of damaged DNA, we induced DNA lesions in vegetative cells using methyl methanesulfonate (MMS) (Wyatt and Pittman, 2006). While the single mutants *psm1^K536A^* and *psm1^K1200A^* were only moderately sensitive or not sensitive to MMS, respectively, double mutant *psm1^K536A K1200A^* was not able to grow in the presence of 0.02% MMS (Fig. 3B). MMS induces a plethora of DNA lesions including those that cause replication forks to arrest (Boddy et al., 2000; Murray et al., 1997; Stewart et al., 1997). To test whether Psm1 residues K536 and K1200 are also required for the restart of collapsed replication forks, we tested the sensitivity of the *psm1^K536A K1200A^* to the topoisomerase I poison camptothecin (CPT). CPT stabilizes topoisomerase I-DNA intermediates leading to the accumulation of DNA breaks and collapse of replication forks at these DNA breaks (Avemann et al., 1988; Strumberg et al., 2000). The *psm1^K536A K1200A^* mutant cells were more sensitive to 2.5 µM CPT than wild-type cells (Fig. 3B). These results show that Psm1 K536 and K1200, and possibly methylation of K536 and K1200, are required to prevent the toxic effects of MMS and CPT.

We next asked whether methylation of the Psm1 K536 is regulated in response to DNA damage caused by MMS. The levels of methylation of Psm1 K536 did not change dramatically in Psm1–Pk immunoprecipitates from cells treated with MMS for up to 120 min (Fig. S2A). However, we cannot exclude the possibility that methylation of the Psm1 K536 is regulated locally, for example at the sites of DNA repair. It remains to be determined if and how Psm1-K536me is regulated.

DNA repair is compromised when cohesin protein levels are decreased (Heidinger-Pauli et al., 2010). However, we found that protein levels of Psm1^K536A K1200A^ were similar to those of wild-type Psm1 (Fig. 2B; Fig. S2A). Therefore, it is unlikely that the sensitivity of *psm1^K536A K1200A^* mutant cells to DNA damaging agents is due to decreased levels of Psm1. The sensitivity of *psm1^K536A K1200A^* mutant cells to MMS and CPT could be caused by the inability of the mutant to efficiently repair the DNA lesions. Although *psm1^K536A K1200A^* mutant cells treated with MMS faithfully segregated chromosomes, and centromeric sister chromatid cohesion was not dramatically altered in metaphase cells (Fig. S2C), we cannot exclude the possibility that sister chromatid cohesion is defective at sites of DNA damage. Furthermore, cohesin also regulates gene expression. Therefore, another possible explanation is that expression of genes required to prevent MMS and CPT toxicity is altered in the *psm1^K536A K1200A^* mutant.

### Genetic interactions between *psm1^K536A K1200A^* and mutants defective in the homologous recombination DNA repair pathway

Both MMS and CPT cause DSBs during DNA replication (Lundin et al., 2005; Tercero et al., 2003). Cohesin accumulates at sites of DSBs where it reinforces sister chromatid cohesion to facilitate repair by homologous recombination (Strom et al., 2004; Unal et al., 2004).

Genetic interactions may reveal functional relationships between genes and pathways. Negative genetic interactions (the phenotype of the double-mutant is stronger than expected) suggest compensatory, redundant pathways, while positive genetic interactions (the phenotype of the double-mutant is less severe than expected) can reflect functions within the same pathway (Ryan et al., 2013). Negative genetic interactions often involve genes with at least partially overlapping functions that can compensate for the absence of each other. While functional interpretation of negative interactions is not straightforward, positive genetic interactions are more interesting because they can provide insight into biochemical relationships between gene products and help define biological pathways. Positive genetic interactions often connect genes encoding members of the same protein complex or linear pathway (Baryshnikova et al., 2013).

**Fig. 4.**
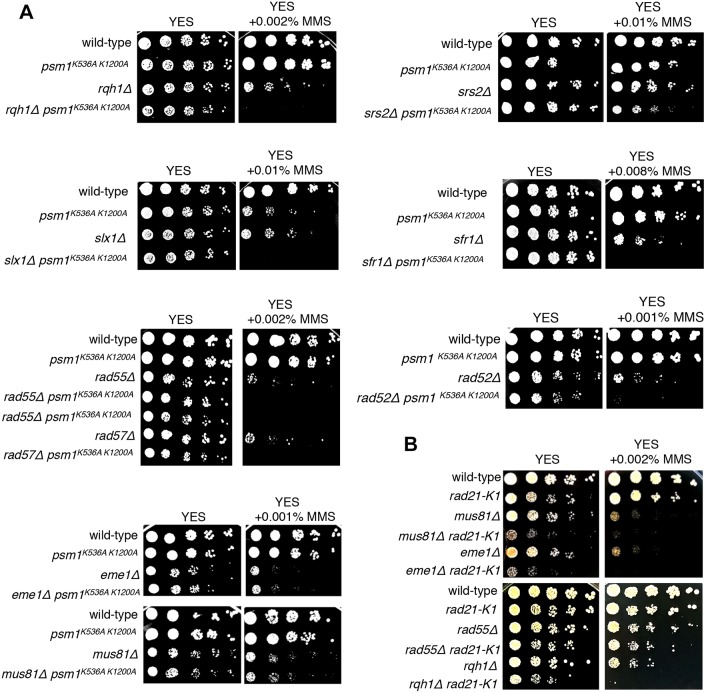
**Genetic interactions between *psm1^K536A K1200A^*, *rad21-K1* and mutations in DNA repair-related genes.** (A) Cells were grown on YES medium for 1 day, diluted in 5-fold steps, spotted onto YES plates containing the indicated amounts of MMS and incubated for 3 days at 30°C. (B) Cells were grown on YES medium for 1 day, diluted in 5-fold steps, spotted onto YES plates containing the indicated amounts of MMS and incubated for 4 days at 25°C.

To better understand the role of Psm1 K536 and K1200, we combined *psm1^K536A K1200A^* with selected mutants defective in homologous recombination. We constructed double mutants between *psm1^K536A K1200A^* and mutations that disrupt recombinational repair of damaged replication forks including *mus81*Δ, *eme1*Δ (the heterodimeric endonuclease complex of the XPF family; Boddy et al., 2001; Doe et al., 2002), *rqh1*Δ (a member of the RecQ family of DNA helicases; Stewart et al., 1997), *srs2*Δ (a UvrD subfamily DNA helicase; Maftahi et al., 2002; Wang et al., 2001), *slx1*Δ (a structure-specific endonuclease, Coulon et al., 2004), and *rad52*Δ, *rad55*Δ, *rad57*Δ and *sfr1*Δ (mediators promoting the formation and/or stabilization of Rad51–ssDNA filaments; Krejci et al., 2012). In the presence of MMS, *psm1^K536A K1200A^* showed negative genetic interactions with most of the tested mutant strains (the phenotype of double-mutant was stronger than the most sensitive of the single mutants) and positive genetic interaction with *mus81*Δ and *eme1*Δ (the phenotype of double mutant was no more sensitive than the most sensitive of the single mutants) (Fig. 4A). Although these results suggest that *psm1^K536A K1200A^ mus81*Δ and *psm1^K536A K1200A^ eme1*Δ double mutants behave differently from other double mutants that we tested, we would like to stress that the *psm1^K536A K1200A^* mutant strain does not show any noticeable sensitivity in the presence of 0.001% MMS and this may affect the results of this assay.

Although in silico modeling suggests that *psm1^K536A^* and *psm1^K1200A^* mutations have only minor effects on the overall structure and electrostatic potential of the Psm1 (data not shown), it is possible that some of the observed phenotypes of *psm1^K536A K1200A^* mutant strains are not due to the absence of methylation but are caused by the replacement of lysine residues by alanine residues. We therefore mutated the K536 and K1200 to arginine residues, which retains a positive charge and is thus considered a conservative mutation (*psm1^K536R K1200R^*). Similar to what was found for the *psm1^K536A K1200A^* mutant, in the presence of MMS, *psm1^K536R K1200R^* showed a negative genetic interaction with *srs2*Δ and *rad55*Δ and a positive genetic interaction with *mus81*Δ (Fig. S3A,B). This suggests that the phenotype of the *psm1^K536A K1200A^* mutant described above is not simply due to the elimination of the positive charge caused by replacing K536 and K1200 with alanine residues. However, the *psm1^K536R K1200R^* mutant strain was less sensitive to MMS than *psm1^K536A K1200A^* (data not shown). Further studies are required to show whether and to what extent the absence of K536 and K1200 methylation contributes to the *psm1^K536A K1200A^* mutant phenotype.

To determine whether the described genetic interactions can be observed with other cohesin mutations that reduce cohesin activity, we tested a hypomorphic cohesin mutation *rad21-K1* (Tatebayashi et al., 1998). Unlike for the *psm1^K536A K1200A^* and *psm1^K536R K1200R^* mutations, *rad21-K1* showed negative genetic interactions with both *mus81*Δ and *eme1*Δ (Fig. 4B). We conclude that the observed pattern of genetic interactions between *psm1^K536A K1200A^* and *psm1^K536R K1200R^* mutants and recombination-deficient mutants is not observed in *rad21-K1* mutant and it is unlikely to be solely due to reduced cohesin activity.

We conclude that the *psm1^K536A K1200A^* mutant shows synthetic phenotypes with mutants defective in the homologous recombination DNA repair pathway, consistent with the notion that Psm1 residues K536 and K1200, and possibly their methylation, are required for efficient repair of damaged DNA. The positive genetic interactions between *psm1^K536A K1200A^* mutant and mutations in genes encoding Mus81-Eme1 endonuclease complex are consistent with the notion that *psm1^K536A K1200A^* mutant negatively affects Mus81–Eme1-dependent DNA repair pathway. We speculate that Psm1 residues K536 and K1200, and possibly methylation of K536 and K1200, are important for an efficient repair of stalled or collapsed replication forks by Mus81–Eme1 complex. Alternatively, Mus81–Eme1 may be required for the function or recruitment of the cohesin to sites of DNA damage. A recent study showed that the Mus81-related endonuclease ERCC1–XPF interacts with cohesin and is required for the recruitment of the CTCF–cohesin complex to promoters and control regions during hepatic development (Chatzinikolaou et al., 2017).

Interestingly, we found that the Psm3 cohesin subunit interacts with Mus81 and that Psc3 weakly interacts with Eme1 in yeast two-hybrid assays (Fig. S4A). We also found one Eme1 peptide in our Rec11–TAP purification but not in other unrelated TAP purifications. Moreover, we found that Mus81–Myc co-immunoprecipitated with Psm1–Pk and the efficiency of co-immunoprecipitation was higher in the presence of MMS (Fig. S4B). This raises the possibility that cohesin physically interacts with the Mus81–Eme1 complex. This interaction may be similar to that described for the mammalian ERCC1–XPF endonuclease and cohesin (Chatzinikolaou et al., 2017). Further investigations will be required to define how the *psm1^K536A K1200A^* mutant functionally associates with Mus81–Eme1 and whether methylation of cohesin affects this interaction.

Taken together, we conclude that cohesin is methylated and that mutations that change the methylated Psm1 K536 and K1200 to alanine affect the Mus81–Eme1-dependent DNA repair pathway. Subunits of the cohesin and Mus81–Eme1 complexes interact in yeast two-hybrid and co-immunoprecipitation assays. Our results are consistent with the notion that methylation of cohesin provides a novel level of regulation that ensures efficient repair of damaged DNA. The Psm1 K536 and K1200 residues and surrounding sequences are conserved in many, but not all, species (Fig. S4C). Thus, our observations on Psm1 methylation are likely to pertain to studies of cohesin and DNA repair in other organisms. Understanding how Psm1 residues K536 and K1200 and their methylation function in DNA repair, and determining how multiple levels of post-translational modifications direct cohesin to function across various cellular processes are important future goals arising from the results described here.

## MATERIALS AND METHODS

### Strain construction

MMS and CPT were obtained from Sigma-Aldrich (cat. no. 129925-25g and C9911-100mg, respectively). Genotypes of strains and the figures and tables in which each was presented is given in Table S1. Mutations were introduced into *psm1* gene using the QuikChangeII kit (Agilent Technologies) as described in Cipak et al., 2011; Gregan et al., 2007.

The following oligonucleotide primers were used for mutagenesis: Psm1K536A, 5′-CTTATGTACTCCTACTCAAAAG**GC**ATATGAAAGCGCAATCGCTGCT-3′; Psm1K536A_antisense, 5′-AGCAGCGATTGCGCTTTCATAT**GC**CTTTTGAGTAGGAGTACATAAG-3′; Psm1 K1200A_sense, 5′-GAAAAATCAGTTGTTCAGT**GC**ATCAGAAGCTTTAGTGGG-3′; Psm1 K1200A_antisense, 5′-CCCACTAAAGCTTCTGAT**GC**ACTGAACAACTGATTTTTC-3′; Psm1K536R_sense, 5′-ATGTACTCCTACTCAAAAGA**G**ATATGAAAGCGCAATCGCTG-3′; Psm1K536R_antisense, 5′-CAGCGATTGCGCTTTCATAT**C**TCTTTTGAGTAGGAGTACAT-3′; Psm1K1200R_sense, 5′-TCATTTCCTTGAAAAATCAGTTGTTCAGTA**G**ATCAGAAGCTTTAGTG-3′; Psm1K1200R_antisense, 5′-CACTAAAGCTTCTGAT**C**TACTGAACAACTGATTTTTCAAGGAAATGA-3′. Bold nucleotides indicate the positions of introduced changes.

Transformants were confirmed by PCR-based analyses and by nucleotide sequencing. The *psm1* gene, either wild-type or carrying mutations, was cloned into the vector pCloneHyg1_Ura4T (p280) using *Bam*HI and *Xma*I restriction enzymes. pCloneHyg1_Ura4T was derived from pCloneHyg1 (GenBank EF101286.1) by reversing the orientation of *hphMX4* and replacing the TEV terminator with that of *ura4* using *Age*I and *Nhe*I restriction enzymes. The resulting pCloneHyg1_Ura4T constructs containing the wild-type or mutated *psm1* gene were digested using *Bam*HI and *Kpn*I enzymes (New England Biolabs). The fragment (∼7.8 kb) containing *psm1* and *hphMX4* was gel purified (Qiagen, Germany), and integrated into diploid yeast strains as described in Gregan et al. (2007). Heterozygous diploid strains were tetrad dissected to isolate haploid strains.

### Induction of synchronous meiosis

Diploid *pat1-114/pat1-114* and *pat1-114/pat1-114 mat-Pc* strains were grown in YES-Ade liquid medium to an optical density at 600 nm (OD_600_)=0.5 at 25°C (∼0.8×10^7^ cells/ml). The cells were collected by centrifugation (3573 ***g*** for 20 min, 25°C), resuspended in EMM2-NH_4_Cl medium and incubated at 25°C for 16–18 h (*pat1-114/pat1-114*) or for 7 h (*pat1-114/pat1-114 mat-Pc*) to arrest cells in G_1_. The cells were resuspended in fresh EMM2-NH_4_Cl medium and induced into meiosis by shifting to 34°C.

To monitor the progression of meiosis, 1 ml of culture was centrifuged (6000 ***g*** for 5 min) and the cell pellet was fixed by adding 0.5 ml of 70% ethanol. Nuclear divisions were monitored by counting the number of DAPI-stained nuclei.

To monitor the pre-meiotic S-phase by flow cytometry, 0.3 ml of the fixed cells was mixed with 3 ml of 50 mM sodium citrate buffer and centrifuged (6000 ***g*** for 5 min) to obtain a pellet. Subsequently, 0.5 ml of 50 mM sodium citrate buffer supplemented with 0.1 mg/ml RNase A (Thermo Scientific) was added to the pellet, and cells were incubated at 37°C for 2 h. This was followed by sonication of the sample twice for 20 s at 40% power with a microtip sonicator (Bandelin Sonoplus HD 2070) to remove cell aggregates. To stain DNA, 0.5 ml of sodium citrate buffer containing 2 μM Sytox Green (Invitrogen) was added and cells were analyzed by FACS (FACS Calibur with Cellquest Pro software) (Cipak et al., 2014).

### TAP purification

Meiotic cultures expressing Rec11–TAP were harvested 2.5–3.5 h after induction of meiosis and cells from 15 l cultures were collected by centrifugation (3573 ***g*** for 20 min, 4°C). Yeast cell powder was made from frozen pellets using a SamplePrep 6870 Freezer Mill (SPEX). Proteins were extracted using IPP150 buffer (IPP150, 50 mM Tris-HCl pH 8.0, 150 mM NaCl, 10% glycerol and 0.1% NP-40) containing complete protease inhibitors (Roche) and 1 mM PMSF (Sigma). Washing steps were performed in Poly-Prep columns (Bio-Rad) by gravity flow. IgG Sepharose™ 6 Fast Flow beads (500 μl; GE Healthcare) were washed with IPP150 buffer, mixed with protein extract, and rotated for 2 h at 4°C. Beads were washed with IPP150 buffer and then with TEV cleavage buffer (TCB; 10 mM Tris-HCl pH 8.0, 150 mM NaCl, 10% glycerol, 0.1% NP-40, 0.5 mM EDTA and1 mM DTT). Protein cleavage was performed in 2 ml of TCB buffer supplemented with 400 units of AcTEV protease (Life Technologies) for 2 h at 16°C. The eluate (2 ml) was supplemented with 6 μl of 1 M CaCl_2_ and mixed with 6 ml of calmodulin binding buffer 1 (CBB1; 10 mM Tris-HCl pH 8.0, 150 mM NaCl, 10% glycerol, 0.1% NP-40, 1 mM imidazole, 1 mM Mg-Acetate, 2 mM CaCl_2_ and 10 mM β-mercaptoethanol). Calmodulin Sepharose 4B beads (150 μl; GE Healthcare) were washed with CBB1 buffer, added to a mixture of eluate and CBB1 buffer, and incubated for 2 h at 4°C. The beads were washed with CBB1 and calmodulin binding buffer 2 (CBB2; 10 mM Tris-HCl pH 8.0, 150 mM NaCl, 1 mM Mg-Acetate, 2 mM CaCl_2_ and 1 mM β-mercaptoethanol). The proteins were eluted using one bed volume of elution buffer (EB; 10 mM Tris-HCl pH 8.0, 150 mM NaCl, 1 mM Mg-acetate, 2 mM EGTA and 1 mM β-mercaptoethanol). A portion of the eluted proteins was separated by 8% SDS-PAGE and silver stained (Cipak et al., 2009). The remaining eluate was analyzed by mass spectrometry (see below).

### Mass spectrometry analysis

#### NanoLC-MS analysis

The nano-high-pressure liquid chromatography (HPLC) system used was an UltiMate 3000 HPLC RSLC nano system (Thermo Fisher Scientific, Bremen, Germany) coupled to an LTQ Orbitrap Velos mass spectrometer (Thermo Fisher Scientific, Bremen, Germany), equipped with a Proxeon nanospray source (Proxeon, Odense, Denmark). Peptides were loaded onto a trap column (Thermo Fisher Scientific, Bremen, Germany, PepMap C18, 5 mm×300 μm internal diameter, 5 μm particles, 100 Å pore size) at a flow rate of 25 μl min^−1^ using 0.1% trifluoroacetic acid (TFA) as a mobile phase. After 10 min, the trap column was switched in line with the analytical column (Thermo Fisher Scientific, Bremen, Germany, PepMap C18, 500 mm×75 μm internal diameter, 3 μm, 100 Å). Peptides were eluted using a flow rate of 230 nl min^−1^, and a binary 2 h gradient, respectively, for 165 min.

The gradient starts with the mobile phases: 98% A (water and formic acid, 99.9:0.1, v/v) and 2% B (water, acetonitrile and formic acid, 19.92:80:0.08, v/v/v) increasing to 35% B over the next 120 min, followed by a gradient in 5 min to 90% B, staying there for 5 min and decreasing in 5 min back to the gradient 98% A and 2% B for equilibration at 30°C.

The LTQ Orbitrap Velos was operated in data-dependent mode, using a full scan in the Orbitrap (*m*/*z* range 350–2000, nominal resolution of 60,000, target value 1E6) followed by tandem mass spectrometry (MS/MS) scans of the 12 most-abundant ions in the linear ion trap. MS/MS spectra (normalized collision energy 35%; activation value q 0.25; activation time 10 ms; isolation width 2, target value 1E4) were acquired, and subsequent activation was performed on fragment ions through multistage activation. The neutral loss mass list was therefore set to −98, −49, and −32.6 *m*/*z*. Precursor ions selected for fragmentation (charge state 2 and higher) were put on a dynamic exclusion list for 90 s. Additionally, singly charged parent ions were excluded from selection for MS/MS experiments and the monoisotopic precursor selection feature was enabled.

#### Data analysis

For peptide identification, the .RAW files were loaded into Proteome Discoverer (version 1.4.0.288, Thermo Scientific). All hereby created MS/MS spectra were searched using Mascot 2.2.07 (Matrix Science, London, UK) against the *Schizosaccharomyces pombe* protein sequence database. The following search parameters were used: β-methylthiolation on cysteine was set as a fixed modification, oxidation on methionine and mono-methylation on lysine and arginine were set as variable modifications. Monoisotopic masses were searched within unrestricted protein masses for tryptic, chymotryptic and no enzymatic specificity. The peptide mass tolerance was set to ±5 ppm and the fragment mass tolerance to ±0.5 Da. The maximal number of missed cleavages was set to 2. The localization of the methylation sites within the peptides was performed with the tool phosphoRS (Taus et al., 2011). Additionally, all of the identified methylation sites were validated by manual inspection of the corresponding MS spectra.

For better visualization, the results of the searches were loaded into Scaffold (version 3.3.1, Proteome Software Inc.), using a minimum of two unique peptides per protein and a Mascot Score of at least 10 as cutoff filters.

### Preparation of anti-Psm1-K536me antibodies

To analyze the methylation status of Psm1, anti-Psm1-K536me antibodies were generated using methylated peptide [CTPTQKK(me)YESAIA] spanning the Psm1 methylation site K536me. Modified and unmodified peptides were synthesized, and rabbits were injected with modified peptide coupled with keyhole limpet hemocyanin carrier by Eurogentec (Belgium). Antibodies against modified and unmodified peptides were subsequently affinity purified in two steps from the crude sera: first by applying the serum to a column containing modified peptide, and second by applying the eluates from the first column to a column containing unmodified peptide. One volume of glycerol was added to the purified antibodies and aliquots were stored at −20°C.

### Detection of Psm1 methylation

The Pk9-tagged Psm1 was immunoprecipitated from cell extracts using anti-Pk antibody-conjugated agarose (Sigma-Aldrich), and analyzed by immunoblotting with anti-Pk and anti-Psm1-K536me antibodies (see below).

### Immunoprecipitation

Exponentially growing cells were harvested and the pellet was resuspended in an equal volume of lysing buffer (50 mM Tris-HCl pH 8.0, 150 mM NaCl, 10% glycerol and 2 mM MgCl_2_) supplemented with 0.1% NP-40 and protease and phosphatase inhibitors [50 mM NaF, 5 mM Na_4_P_2_O_7_, 0.1 mM Na_3_VO_4_, 1 mM β-glycerophosphate, 1 mM PMSF and Roche cOmplete Mini Protease tablets (one tablet per 10 ml of buffer)]. The suspension was frozen in liquid nitrogen and ground into a powder with a SPEX SamplePrep 6870 freezer mill. 1 ml of ice-cold lysing buffer (plus protease inhibitors) was added to 1 g of the frozen powder and gently thawed on ice. 375 units of benzonase (Novagen, 250 U/µl; Merck) was added, and the mixture was rotated at 4°C for 1 h. The lysate was then clarified by spinning at 21,130 ***g*** at 4°C. This was followed by measurement of protein concentration in the clear lysate with a Bradford protein assay (Biorad). Lysate containing 10 mg of total protein was added to 50 µl of packed anti-V5 agarose beads that were prepared according to manufacturer's instructions (Sigma-Aldrich) and incubated overnight at 4°C. Next day, the sample was spun at 5000 ***g*** for 30 s and washed five times with lysing buffer (plus protease inhibitors). Bound proteins were eluted twice with 50 µl 4× SDS loading buffer. The input and eluates were analyzed by western blotting as described below.

### Co-immunoprecipitation

Cells expressing Psm1–Pk, Mus81–Myc or both Psm1–Pk and Mus81–Myc were grown in YES or YES plus 0.01% MMS for 3 h. Protein extracts were prepared as described above for TAP purification. Protein extracts were incubated with anti-V5 agarose beads for 4 h to bind Psm1-Pk. Eluted proteins were separated (8% SDS-PAGE) and transferred to a PVDF membrane (Immobilon-P, 0.45 μm, Millipore). The membrane was blocked with 2% milk in PBS with 0.05% Tween 20 (PBST) and probed with primary antibodies against the Pk and Myc tags. To detect Psm1–Pk, we used monoclonal anti-V5 antibody, diluted 1:5000 (V8012, Sigma-Aldrich) and secondary antibody goat anti-mouse-IgG conjugated to horseradish peroxidase (HRP), diluted 1:20,000 (sc-2005, Santa Cruz Biotechnology). To detect Mus81–Myc, we used rabbit c-Myc antiserum, diluted 1:10,000 (CM-100, Gramsch, Germany) and secondary antibody mouse anti-rabbit-IgG conjugated to HRP, diluted 1:20,000 (sc-2357, Santa Cruz Biotechnology). The ratio of loading between input and immunoprecipitated Pk samples was ∼1:30.

### Western blot analysis

Proteins were separated by electrophoresis through 10% polyacrylamide gels containing SDS (0.1%) and transferred onto a PVDF membrane (Immobilon-P membrane with 0.45 µm pore size from Millipore). The membrane was blocked with 2% milk PBST and probed with primary antibodies. Psm1-K536me was detected using rabbit anti-Psm1-K536me, diluted 1:100 (custom made by Eurogentec, UK) and goat anti-rabbit IgG conjugated HRP secondary antibody, diluted 1:5000 (Jackson Immunoresearch). Psm1–Pk9 was detected using mouse-anti-Pk (V5) antibody, diluted 1:1000 (MCA1360 Serotec) and rabbit anti-mouse IgG conjugated to HRP secondary antibody, diluted 1:10,000 (Santa Cruz Biotechnology). Approximate positions of molecular mass markers are indicated in figures.

### Dot blot assay

For dot blot analysis, peptides CTPTQKK(me)YESAIA, CTPTQKKYESAIA and CTPTQKAYESAIA were dissolved in water at room temperature to a final concentration of 100 µg/µl and spotted on a methanol activated PVDF membrane (Immobilon-P membrane, 0.45 µm pore size from Millipore). The membrane was blocked with 2% milk in PBST and probed with pre-immune serum or anti-Psm1-K536me antibody (1:100) in 2% milk in PBST. Goat anti-rabbit-IgG conjugated to HRP was used as secondary antibody, diluted 1:5000 (Jackson Immunoresearch).

### Chromatin spreads

Chromatin spreads were prepared as described in Loidl and Lorenz (2009). 6 ml of exponentially growing cells were harvested and resuspended in 1 ml of spheroplasting solution (0.65 M KCl with 10 mM dithiothreitol) including 20 µl zymolyase (10 mg/ml; AMS Biotechnology), 20 µl glucuronidase (0.4%; Sigma-Aldrich), 60 µl lysing enzymes (250 mg/ml; L1412, Sigma-Aldrich) and digested at 30°C until lysis of spheroplasts was observed in 1% sodium sarcosyl. The reaction was stopped by adding 9 ml of ice-cold stop solution [0.1 M 2-(N-morpholino) ethane sulfonic acid (MES), 1 mM EDTA, 0.5 mM MgCl_2_, 1 M sorbitol, pH 6.4]. The cells were collected by centrifugation (720 ***g*** for 4 min) and resuspended in 1 ml of stop solution. 20 µl of the cell suspension was transferred on an ethanol-cleaned slide and prefixed with 40 µl of fixative (4% paraformaldehyde, 3.6% sucrose), lysed with 80 µl of detergent (1% lipsol) and the mixture was spread with 80 µl of fixative. The air-dried slides were washed with PBS, and blocked for 30 min with 50 µl of blocking solution (1% BSA in PBS). To detect Psm1-K536me, the slides were incubated overnight with 30 µl of anti-Psm1-K536me antibody (Eurogentec, 1:50 dilution in PBS) at 4°C in a humid chamber. Slides were washed with 10 ml of PBS (10 min, room temperature) and incubated with 30 µl of goat anti-rabbit-IgG antibody, diluted 1:500 (Life Technologies) for 2 h at room temperature. Slides were washed with 10 ml of PBS (10 min, room temperature) and mounting medium with DAPI (Vectashield) was used for microscopy. Images were taken using the following settings: exposure time, FITC, 3154.4 ms, DAPI, 2924.6 ms; acquisition mode, live speed slow; resolution, 1388×1040; binning, 1×1; pixel size, 6.45×6.45 μm.

### Yeast two-hybrid analysis

Full-length coding regions for Psm1, Psm3, Psc3, Rad21, Mus81 and Eme1 were amplified from cDNA with primers that added a 5′ *Sfi*I and 3′ *Sma*I or *Bam*HI (for Fbh1) restriction sites and then cloned into plasmid pGBKT7 (Clontech) for GAL4 DNA-binding domain bait constructs, or into plasmid pGADT7 (Clontech) for GAL4 activation domain prey constructs.

Primers used were 5′-AAAAGGCCATGGAGGCCtatatcacaaaaattgttattcaaggg-3′ and 5′-AAAACCCGGGttatccttcaacgaatgccatagcctcctcc-3′ for Psm3, 5′-AAAAGGCCATGGAGGCCagtgagtctgttactactggatctg-3′ and 5′-AAAACCCGGGttaattcaaaaactcatccacaatagcag-3′ for Psc3, 5′-AAAAGGCCATGGAGGCCttctattcagaggccattctttc-3′ and 5′-AAAACCCGGGtcatagtgatgaaagtagcattccacgtttagc-3′ for Rad21, 5′-AAAAGGCCATGGAGGCCgattgtggaaatccgctgtttttacaat-3′ and 5′-AAAACCCGGGtcaagattctggaaagaaaacgcttgc for Mus81 and 5′-AAAAGGCCATGGAGGCCggacgtttactaagactggaagttg-3′ and 5′-AAAACCCGGGttactccacataaccttccaagttgattgataatgttcgtgaagaattctcc-3′ for Psm1 and 5′-AAAAGGCCATGGAGGCCactctgcagtctacagattccgc-3′ and 5′-AAAACCCGGGctatggtgcatcactggatgcttctttacataag-3′ for Eme1. Lowercase letters represent the gene-specific part of the oligos. *mus81* and *eme1* were cloned into pGADT7 (plasmid numbers p362 and p360, respectively) while *psm1*, *psm3*, *psc3* and *rad21* were cloned into pGBKT7 plasmid (plasmid numbers p351, p353, p356 and p357, respectively). All inserts were verified by sequencing. Bait and prey plasmids were introduced into *S. cerevisiae* strain PJ69-4A (Clontech) by standard lithium acetate-mediated transformation. Transformants were tested for bait–prey interaction by spotting onto SD minimal medium lacking appropriate amino acids according to the manufacturer's instructions. We performed at least two independent transformations and growth tests.

## Supplementary Material

Supplementary information
