# Supplementary material for: Mutations that prevent methylation of cohesin render sensitivity to DNA damage in S. pombe
Source: J Cell Sci. 2018 Jul 6;131(13):jcs214924. doi: 10.1242/jcs.214924 (PMC6051343; doi:10.1242/jcs.214924)
Supplement: Supplementary information [file joces-131-214924-s1.pdf]

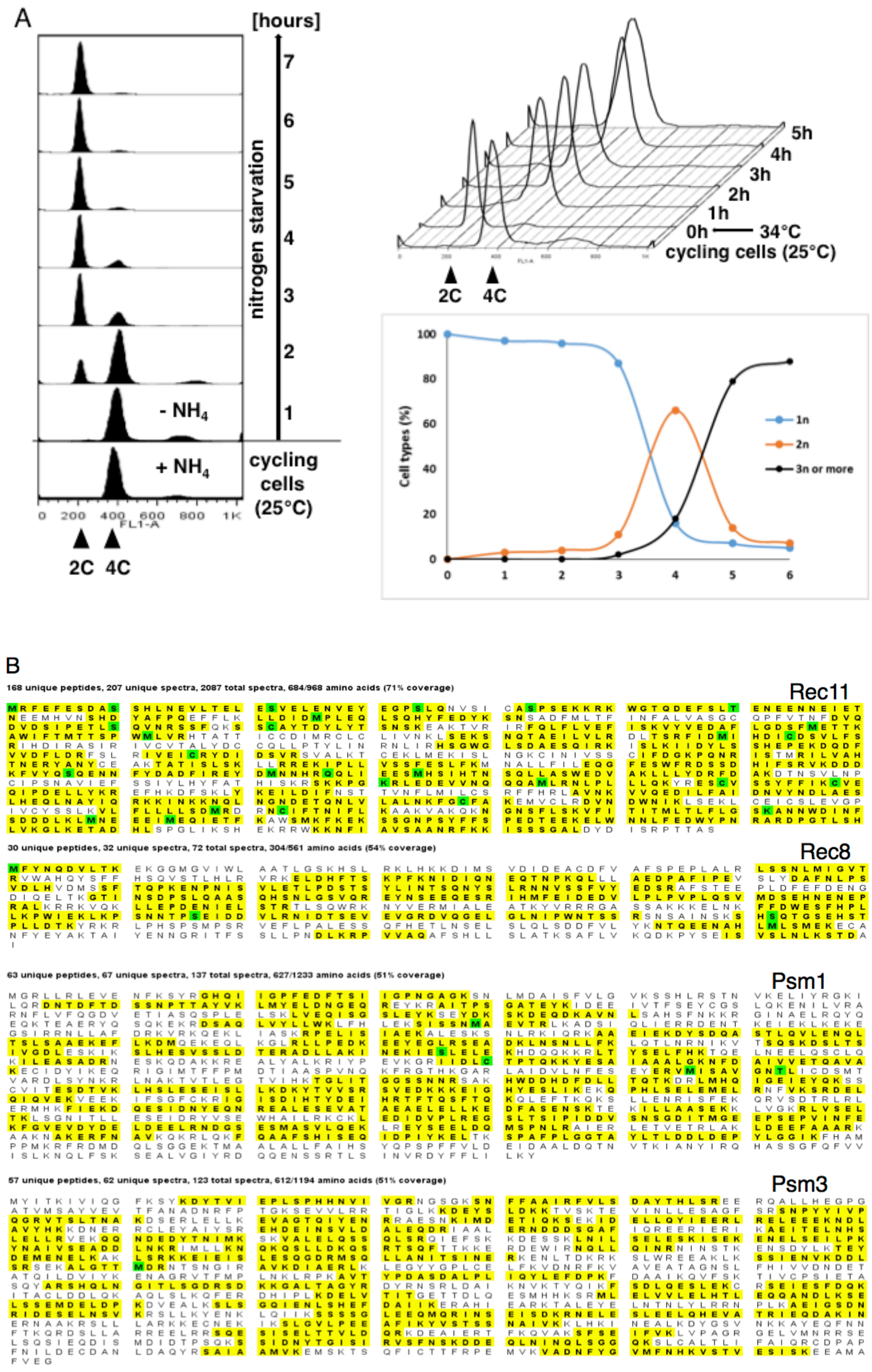
A
[hours]
nitrogen starvation
7
6
5
4
3
2
1
- NH4
+ NH4
cycling cells (25°C)
2C 4C
FL1-A
5h
4h
3h
2h
1h
0h
34°C
cycling cells (25°C)
Cell types (%)
1n
2n
3n or more
B
168 unique peptides, 207 unique spectra, 2087 total spectra, 684/968 amino acids (71% coverage)
Rec11
30 unique peptides, 32 unique spectra, 72 total spectra, 304/561 amino acids (54% coverage)
Rec8
63 unique peptides, 67 unique spectra, 137 total spectra, 627/1233 amino acids (51% coverage)
Psm1
57 unique peptides, 62 unique spectra, 123 total spectra, 612/1194 amino acids (51% coverage)
Psm3

Figure S1 Progression of *pat1*-induced meiosis and mass-spectrometry analysis of cohesin subunits.

(A) *pat1-114/pat1-114 mat-Pc* cells expressing Rec11-TAP were arrested by nitrogen starvation and released into meiosis at 34°C by inactivation of Pat1 (as described in Fig. 2A). Cells were harvested at the indicated time points (hours), fixed with ethanol and analysed by flow-cytometry. In parallel, fixed cells were stained with DAPI and nuclei were counted in 100 cells per time point. Shown are the fractions of cells that contained one nucleus (1n), two nuclei (2n) or more than two nuclei (3n or more) at the indicated time points.

(B) Cohesin subunits associated with Rec11-TAP were isolated and analysed by mass-spectrometry as described in Figure 1. Sequence coverage is indicated in yellow.

Journal of Cell Science • Supplementary information

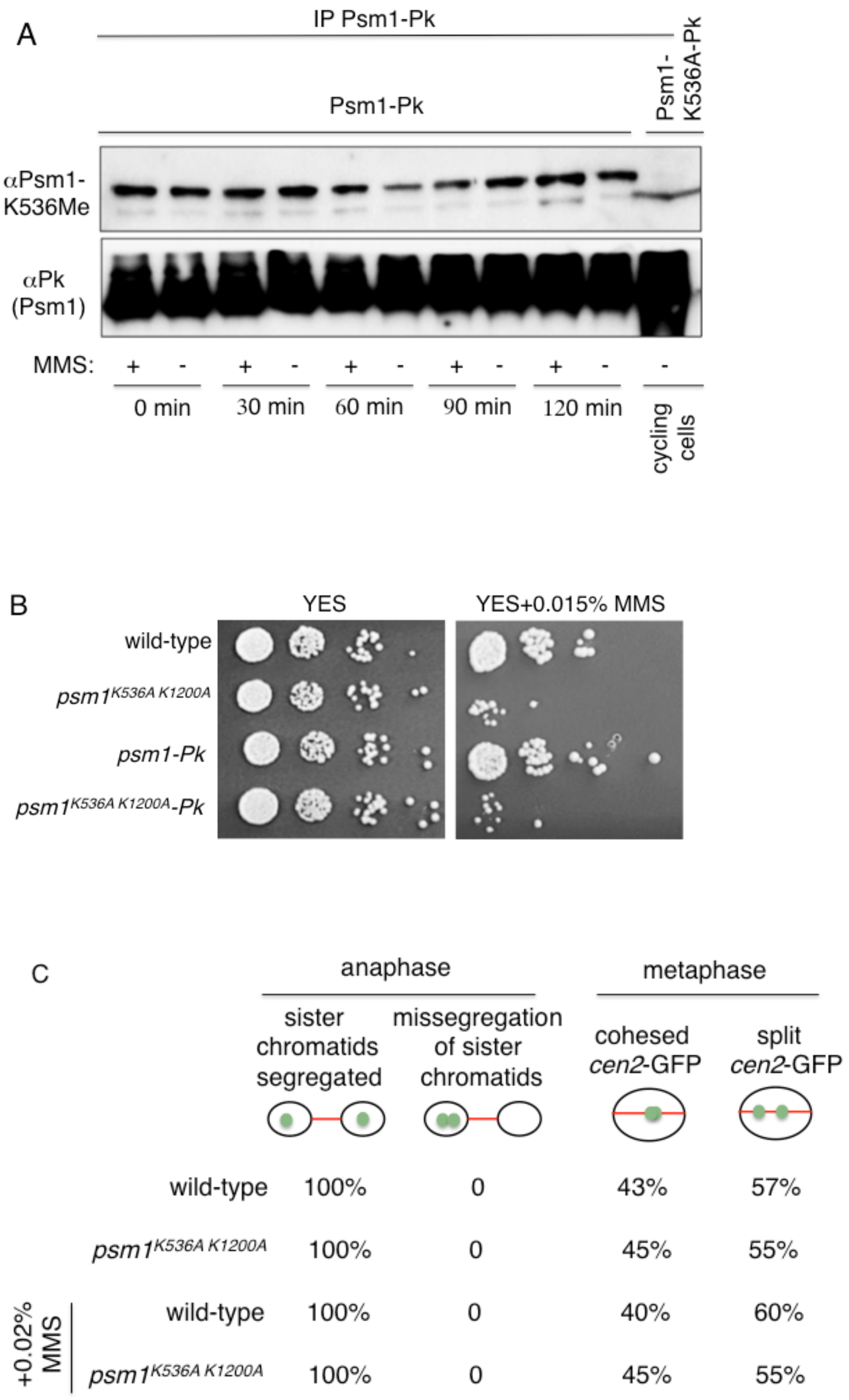

Journal of Cell Science • Supplementary information

Figure S2. The levels of Psm1-K536me do not change in response to MMS treatment, Psm1-Pk is functional and chromosome segregation during mitosis is normal in *psm1*$^{K536A\ K1200A}$ mutant cells.

(A) Psm1-Pk protein was immunoprecipitated (IP) from the indicated time points after adding 0.01% MMS (10 mg of total proteins were applied to 50 μg of packed beads). Samples were analysed by Western blotting using anti-Psm1-K536me and anti-Pk antibodies. Cycling cells expressing Psm1-K536A-Pk were used as a negative control.

(B) Sensitivity of cells expressing Psm1-Pk and Psm1$^{K536A\ K1200A}$-Pk to methyl methanesulfonate (MMS). Serial dilutions of wild-type, *psm1*$^{K536A\ K1200A}$, *psm1-Pk* and *psm1*$^{K536A\ K1200A}$*-Pk* strains were spotted on YES containing or lacking the MMS and grown for 2 days at 32°C. Psm1-Pk is functional as assayed by this MMS-sensitivity test.

(C) Segregation of chromosome 2 was scored in a wild-type and *psm1*$^{K536A\ K1200A}$ strains carrying chromosome 2 marked by *cen2*-GFP. Strains were grown at 32°C for 3 hours in YES medium with or without 0.02% MMS. Cells were fixed and immunostained for tubulin and GFP. DNA was visualized by DAPI staining. At least 100 anaphase or metaphase cells were examined under the fluorescence microscope.

Journal of Cell Science • Supplementary information

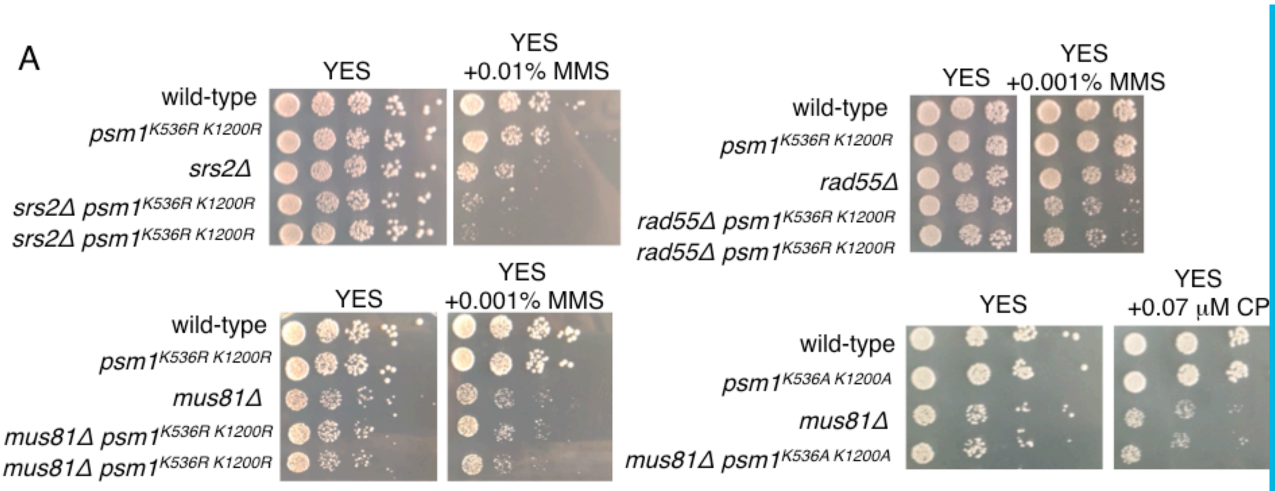

B

	% plating efficiency (± standard deviation)			
	**YES**	**YES +0.001% MMS**	**YES +0.002% MMS**	**YES +0.004% MMS**
**wild-type**	62.9 ± 9.7	62.6 ± 4.1	60.8 ± 5.6	61.8 ± 5.0
***psm1*$^{K536A\ K1200A}$**	80.6 ± 2.7	78.1 ± 4.0	83.7 ± 4.4	78.7 ± 4.4
***psm1*$^{K536R\ K1200R}$**	72.5 ±3.2	72.7 ± 3.1	71.1 ± 6.6	71.6 ± 4.1
mus81Δ	40.4 ± 2.6	13.5 ± 2.6	0.6 ± 0.2	-
***mus81Δ psm1*$^{K536A\ K1200A}$**	35.3 ± 2.0	12.3 ± 0.9	0.5 ± 0.3	-
***mus81Δ psm1*$^{K536R\ K1200R}$**	40.3 ± 3.6	12.1 ± 2.2	0.3 ± 0.2	-
sfr1Δ	68.4 ± 9.2	-	67.2 ± 5.2	65.1 ± 6.4
***sfr1Δ psm1*$^{K536A\ K1200A}$**	75.1 ± 3.2	-	72.6 ± 8.7	2.6 ± 1.3

Journal of Cell Science • Supplementary information

Figure S3. Genetic interactions between *psm1*$^{K536RA\ K1200A}$, *psm1*$^{K536R\ K1200R}$ and mutations in DNA repair-related genes.

(A) Cells were grown on YES medium for one day, diluted in 5-fold steps, spotted onto YES plates containing the indicated amounts of methyl methanesulfonate (MMS) or camptothecin (CPT) and incubated for 3 days at 30°C.

(B) The plating efficiency indicates the percentage of cells plated onto a plate capable of forming colonies. To determine the plating efficiency, the number of cells plated on the freshly prepared YES plates containing the indicated concentrations of MMS was divided by number of colonies formed after 4 days of incubation. Data are means ± standard deviation of at least four independent experiments.

Journal of Cell Science • Supplementary information

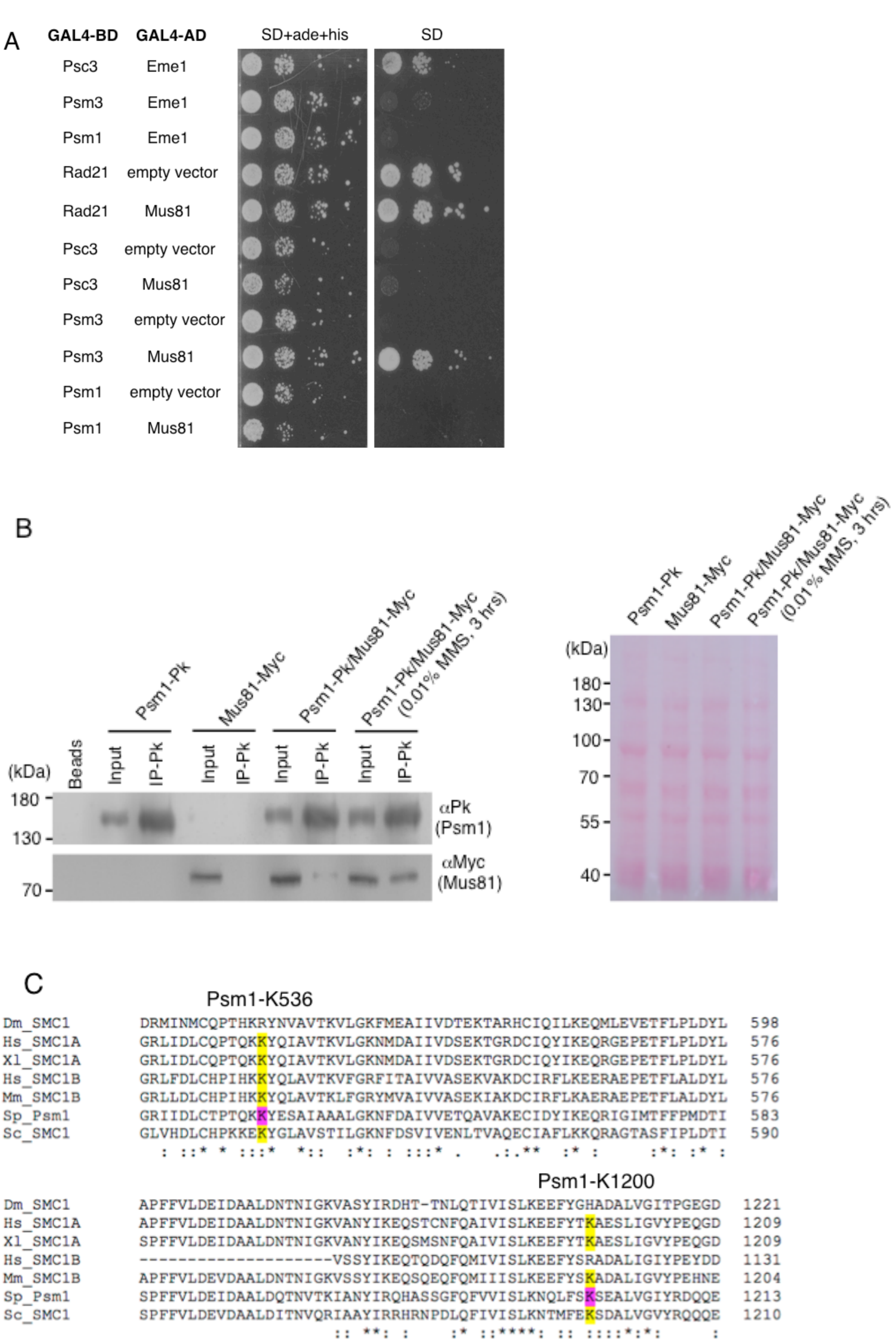

Journal of Cell Science • Supplementary information

Figure S4. Physical interactions between subunits of cohesin and Mus81-Eme1 complexes and conservation of Psm1 lysine residues 536 and 1200 in other organisms.

(A) Strains expressing Mus81 or Eme1 fused to the GAL4 transcription activation domain and Psm1, Psm3, Psc3 or Rad21 fused to the GAL4 DNA-binding domain were spotted at 10-fold serial dilutions on SD plates or SD plates supplemented with adenine and histidine (SD+ade+his). The empty vector pGADT7 containing GAL4 transcription activation domain was used as a negative control. Growth of the strain expressing Psm3 fused to the GAL4 DNA-binding domain and Mus81 fused to the GAL4 transcription activation domain on the plate without histidine and adenine (SD) indicates interaction between Psm3 and Mus81. A weak interaction between Psc3 and Eme1 was also detected. The interaction between Rad21 and Mus81 is a false positive because the control strain carrying Rad21 fused to the GAL4 DNA-binding domain and an empty vector containing GAL4 transcription activation domain was also able to grow on the plate without histidine and adenine (SD).

(B) Protein extracts were prepared from cycling cells expressing Psm1-Pk, Mus81-Myc or both Psm1-Pk and Mus81-Myc grown in YES or YES+0.01% MMS, as indicated. Proteins bound to anti-V5 agarose beads, which bind the Pk tag on Psm1, were analyzed for Mus81 by Western blotting with anti-Myc antibody. Ponceau staining of the membrane after protein transfer shows protein amounts loaded in inputs.

(C) ClustalW generated alignment of Smc1 protein sequences from *Drosophila melanogaster* (Dm_SMC1), human (Hs_SMC1A, Hs_SMC1B)), *Xenopus laevis* (Xl_SMC1A), mouse (Mm_SMC1B), *S. pombe* (Sp_Psm1) and *S. cerevisiae* (Sc_SMC1). Positions of Psm1 lysines K536 and K1200 are indicated.

Journal of Cell Science • Supplementary information

Table S1. *S. pombe* strains

Strain	Genotype	Used in Figure
JG16384	*h^-/h^- ade6-M210/ade6-M216 pat1-114/pat1-114 Rec11-TAP::kanMX6/Rec11-TAP::kanMX6 mat-Pc::lys1*	1, S1
JG17622	*h^- ade6-210 psm1::psm1-pk9::kanMX6*	2B, S2A, S2B
JG17624	*h^- ade6-210 psm1::psm1-K536A K1200A-pk9::kanMX6*	2B, S2A, S2B
JG17680	*h^+/h^- ade6-M210/ade6-M216 psm1+/psm1::psm1-K536A K1200A-hphMX4*	3A
JG16543	*h^+/h^- ade6-M210/ade6-M216 psm1+/psm1::natMX4 lys1-37/lys1+*	3A
JG17331	*h^+ ade6-M216 lys1-37*	2C, 3, 4, S3
JG17184	*h^+ psm1::psm1-K536A::hphMX4*	3B
JG17187	*h^- psm1::psm1-K1200A::hphMX4*	3B
JG17189	*h^- psm1::psm1-K536A K1200A::hphMX4*	2C, 3, 4, S3, S2B
JG17179	*h^+ psm1::psm1$^+$::hphMX4*	3B
JG17543	*h^{90} rqh1::kanMX4*	4A
JG17879	*psm1::psm1-K536A K1200A::hphMX4 rqh1::kanMX4*	4A
JG17792	*h^{90} srs2::kanMX4*	4A, S3
JG17875	*psm1::psm1-K536A K1200A::hphMX4 srs2::kanMX*	4A
JG17827	*h^{90} eme1::kanMX4*	4A
JG17869	*psm1::psm1-K536A K1200A::hphMX4 eme1::kanMX4*	4A
JG17884	*h^{90} mus81::kanMX4*	4A, S3
JG17924	*h^- psm1::psm1-K536A K1200A::hphMX4 mus81::kanMX4*	4A, S3
JG17551	*h^+ ade6-52 slx1::kanMX6*	4A
JG17877	*psm1::psm1-K536A K1200A::hphMX4 slx1::kanMX6*	4A
JG17468	*h^+ ura4-D18 leu1-32 rad57::kanMX4*	4A
JG17863	*h^- psm1::psm1-K536A K1200A::hphMX4 rad57::kanMX4*	4A
JG17469	*h^+ ura4-D18 leu1-32 rad55::kanMX4*	4A, S3
JG17861	*psm1::psm1-K536A K1200A::hphMX4 rad55::kanMX4 clone 1*	4A
JG17862	*psm1::psm1-K536A K1200A::hphMX4 rad55::kanMX4 clone 2*	4A
JG17823	*h^+ ura4-D18 leu1-32 rad52::kanMx4*	4A
JG17865	*h^- psm1::psm1-K536A K1200A::hphMX4 rad52::kanMX4*	4A
JG17746	*h^{90} sfr1::natMX4*	4A, S3
JG17871	*psm1::psm1-K536A K1200A::hphMX4 sfr1::natMX4*	4A, S3
JG17680	*h^-/h^+ ade6-M210/ade6-M216 psm1+/psm1::psm1-K536A K1200A::hphMX4*	3A
JG16543	*h^-/h^+ ade6-M210/ade6-M216 psm1+/psm1::natMX4*	3A
JG16539	*h^-/h^+ ade6-M210/ade6-M216*	3A
JG18082	*psm1::psm1-K536R K1200R::hphMX4 mus81::kanMX4 clone1*	S3
JG18083	*psm1::psm1-K536R K1200R::hphMX4 mus81::kanMX4 clone2*	S3
JG18092	*psm1::psm1-K536R K1200R::hphMX4 srs2::kanMX4 clone1*	S3
JG18093	*psm1::psm1-K536R K1200R::hphMX4 srs2::kanMX4 clone2*	S3
JG18096	*psm1::psm1-K536R K1200R::hphMX4 rad55::kanMX4 clone1*	S3
JG18097	*psm1::psm1-K536R K1200R::hphMX4 rad55::kanMX4 clone2*	S3
JG18168	*h^- psm1::psm1-K536R K1200R::hphMX4*	S3
JG17214	*h^{90} ade6-216 ura4-D18 cen2(D107)[:: kanr-ura4+-lacOp] his7+::lacI-GFP psm1::phygUra4Tpsm1+*	S2C
JG17254	*h^+ cen2(D107):Kan-ura4+-lacO his7+::lacI-GFP psm1::pHygUra4T psm1K536A K1200A*	S2C
JG11363	*h^+ rad21-K1-ura4+ leu1-32 ura4D18 ade6-210*	4B
JG18180	*h^- rad21-K1-ura4+ leu1-32 ura4D18 ade6-210*	4B
JG18178	*rad21-K1 mus81::kanMX4*	4B
JG18182	*rad21-K1 eme1::kanMX4*	4B
JG18183	*rad21-K1 rad55::kanMX4*	4B
JG18184	*rad21-K1 rqh1::kanMX4*	4B
JG18140	*h^+ ade6-M210 psm1-pk9::kanMX4*	S4B
JG18186	*h^- leu1 ura4 mus81-myc::kanMX6*	S4B
JG18196	*psm1-pk9::kanMX4 mus81-myc::kanMX6*	S4B

(other auxotrophic markers have not been scored)

Journal of Cell Science • Supplementary information
